# *UCHL1 * S18Y variant is a risk factor for Parkinson’s disease in Japan

**DOI:** 10.1186/1471-2377-12-62

**Published:** 2012-07-28

**Authors:** Yoshihiro Miyake, Keiko Tanaka, Wakaba Fukushima, Chikako Kiyohara, Satoshi Sasaki, Yoshio Tsuboi, Tatsuo Yamada, Tomoko Oeda, Hiroyuki Shimada, Nobutoshi Kawamura, Nobutaka Sakae, Hidenao Fukuyama, Yoshio Hirota, Masaki Nagai

**Affiliations:** 1 Department of Preventive Medicine and Public Health, Faculty of Medicine, Fukuoka University, Fukuoka, Japan; 2 Department of Public Health, Osaka City University Graduate School of Medicine, Osaka, Japan; 3 Department of Preventive Medicine, Graduate School of Medical Sciences, Kyushu University, Fukuoka, Japan; 4 Department of Social and Preventive Epidemiology, School of Public Health, The University of Tokyo, Tokyo, Japan; 5 Department of Neurology, Faculty of Medicine, Fukuoka University, Fukuoka, Japan; 6 Department of Neurology, Clinical Research Institute, Utano National Hospital, Kyoto, Japan; 7 Department of Geriatrics and Neurology, Osaka City University Graduate School of Medicine, Osaka, Japan; 8 Department of Neurology, Neurological Institute, Graduate School of Medical Sciences, Kyushu University, Fukuoka, Japan; 9 Human Brain Research Center, Kyoto University Graduate School of Medicine, Kyoto, Japan; 10 Department of Public Health, Saitama Medical University Faculty of Medicine, Saitama, Japan; 11 Other members of the Study Group are listed in the Appendix

## Abstract

**Background:**

A recent meta-analysis on the *UCHL1 * S18Y variant and Parkinson’s disease (PD) showed a significant inverse association between the Y allele and PD; the individual studies included in that meta-analysis, however, have produced conflicting results. We examined the relationship between *UCHL1 * S18Y single nucleotide polymorphism (SNP) and sporadic PD in Japan.

**Methods:**

Included were 229 cases within 6 years of onset of PD, defined according to the UK PD Society Brain Bank clinical diagnostic criteria. Controls were 357 inpatients and outpatients without neurodegenerative disease. Adjustment was made for sex, age, region of residence, smoking, and caffeine intake.

**Results:**

Compared with subjects with the CC or CA genotype of *UCHL1 * S18Y SNP, those with the AA genotype had a significantly increased risk of sporadic PD: the adjusted OR was 1.57 (95 % CI: 1.06 − 2.31). Compared with subjects with the CC or CA genotype of *UCHL1 * S18Y and the CC or CT genotype of *SNCA* SNP rs356220, those with the AA genotype of *UCHL1 * S18Y and the TT genotype of SNP rs356220 had a significantly increased risk of sporadic PD; the interaction, however, was not significant. Our previous investigation found significant inverse relationships between smoking and caffeine intake and PD in this population. There were no significant interactions between *UCHL1 * S18Y and smoking or caffeine intake affecting sporadic PD.

**Conclusions:**

This study reveals that the *UCHL1 * S18Y variant is a risk factor for sporadic PD. We could not find evidence for interactions affecting sporadic PD between *UCHL1 * S18Y and *SNCA* SNP rs356220, smoking, or caffeine intake.

## Background

The ubiquitin proteasome system regulates the degradation of key regulatory proteins as well as misfolded and damaged proteins [1]. Ubiquitin carboxy-terminal hydrolase L1 (UCHL1) is a 223-amino acid protein that belongs to the family of deubiquitinating enzymes known as UCHL1 − 5. UCHL1 is selectively and abundantly expressed in neurons, constituting 1–2 % of total soluble brain protein [2]. It is known to generate free monomeric ubiquitin from ubiquitin proproteins [3]. UCHL1 activity has been shown to be required for normal synaptic function: pharmacological suppression of UCHL1 significantly reduces monomeric ubiquitin levels and causes alterations in synaptic protein distribution and spine morphology [4].

The *UCHL1 * gene is located on chromosome 4p14. The S18Y variant arises due to a cytosine-to-adenine transversion at codon 18 of exon 3 (rs5030732). Ragland et al. [5] conducted a meta-analysis of published case–control studies concerning the *UCHL1 * S18Y variant and Parkinson’s disease (PD), which indicated a significant inverse association between the Y allele and PD under a recessive model using data from studies in populations of Asian ancestry and a similar significant inverse relationship under a dominant model using data from studies in populations of European ancestry. It is worth noting, however, that the 15 studies included in this meta-analysis [6-20] and another five recent studies that were not included in the meta-analysis [21-25] have produced conflicting results.

Here, we examined the relationship between *UCHL1 * S18Y single nucleotide polymorphism (SNP) and the risk of sporadic PD using data from a multicenter hospital-based case–control study in Japan. Because UCHL1 and α-synuclein proteins co-localized on the halo of Lewy bodies in a patient with sporadic PD [26], we also investigated the possibility of an interaction between *UCHL1 * S18Y SNP and *SNCA* SNP rs356220. Moreover, possible interactions between *UCHL1 * S18Y and smoking or caffeine intake were assessed.

## Methods

### Study population

#### Recruitment of cases

Patients with PD were recruited at 3 university hospitals and 1 national hospital in Fukuoka Prefecture, a metropolitan area of Kyushu Island in southern Japan, and in 3 university hospitals, 3 national hospitals and 1 municipal hospital in Osaka, Kyoto, and Wakayama Prefectures, which are part of the Kinki region, located in the midwestern part of the mainland. Eligible patients were within 6 years of the onset of PD and had been diagnosed by one of the collaborating neurologists according to the United Kingdom PD Society Brain Bank clinical diagnostic criteria [27]. The neurologists in charge asked eligible PD patients to take part in our case–control study. Of 298 eligible PD patients identified during the period between 1 April 2006 and 31 March 2008, 250 agreed to participate in the study (response rate: 84 %).

#### Recruitment of control subjects

During the same period, control subjects without a previous diagnosis of neurodegenerative disease were recruited from departments other than the neurology departments (orthopedic surgery, ophthalmology, otorhinolaryngology, plastic surgery, and oral surgery) at 3 of the 11 collaborating hospitals: 1 university hospital in Fukuoka Prefecture and 1 university hospital and 1 national hospital in the Kinki region. Control subjects were not, individually or in larger groups, matched to cases. Potential control subjects were asked by an attending doctor or by one of our research nurses to participate in our case–control study when they were seen as outpatients or hospitalized at any of these 3 hospitals. Altogether, 372 of the potential control subjects participated in our study whereas 156 refused (response rate: 70 %).

#### The present study population

Of the 250 cases and 372 controls who participated in our study, 240 cases and 371 controls gave informed consent to genotyping. Excluded were 11 cases and 12 controls with a family history of PD, 1 control with missing data on smoking, and 1 control with missing data on caffeine intake, leaving data on 229 cases and 357 control subjects available for analysis. The ethics committees of the 11 collaborating hospitals (Fukuoka University; Utano National Hospital; Osaka City University; Kyushu University; Wakayama Medical University; Kyoto University; Kurume University; Minami-Kyoto National Hospital; Toneyama National Hospital; Kyoto City Hospital; and National Omuta Hospital) approved our case–control study. Written informed consent was obtained from all subjects.

### Questionnaires

Participants filled out a set of 2 self-administered questionnaires and mailed these materials to the data management center or handed them to research nurses. Our research technicians completed missing answers and/or illogical data by telephone or in-person interview.

Dietary habits during the preceding month were assessed using a self-administered, semi-quantitative, comprehensive diet history questionnaire. Reported intake levels of coffee, black tea, and Japanese and Chinese teas, including green tea and oolong tea, were used to estimate caffeine intake. Energy-adjusted intake was calculated according to the density method. A second questionnaire elicited information on sex, age, smoking habits, and family history of PD. A history of smoking was defined as having smoked at least once per day for at least one year.

### DNA extraction and genotyping

The attending physicians or our research nurses collected buccal specimens with BuccalAmp swabs (Epicenter BioTechnologies, Madison, WI, USA). Genomic DNA was extracted using a BuccalAmp DNA extraction kit (Epicenter BioTechnologies). Genotyping was performed using a TaqMan SNP Genotyping Assay on a StepOnePlus machine, according to the manufacturer’s instructions (Applied Biosystems, Foster City, CA, USA).

### Statistical analysis

We tested agreement with Hardy-Weinberg equilibrium among the control subjects using the chi-square test. Logistic regression analysis was applied to estimate crude odds ratios (ORs) and 95 % confidence intervals (CIs) of sporadic PD in relation to *UCHL1 * S18Y SNP. Multiple logistic regression analysis was used to adjust for sex, age, region of residence, smoking, and caffeine intake. *SNCA* SNP rs356220, but not SNPs rs356229, rs356219, rs7684318, or rs2736990 under a recessive model [28], smoking [29], and caffeine intake [30] were significantly associated with the risk of PD in this population. We examined multiplicative and additive interactions between *UCHL1 * S18Y and *SNCA* SNP rs356220, smoking, or caffeine intake with regard to the risk of sporadic PD. Multiplicative interaction was estimated by introducing a multiplicative term into a multiple logistic regression model. Three measures for the additive interaction were calculated using the Excel sheet provided by Andersson et al. [31]: 1) relative excess risk due to interaction (RERI), 2) attributable proportion due to interaction (AP), and 3) synergy index (S). RERI is the excess risk due to an interaction relative to the risk without exposure. AP refers to the attributable proportion of disease among individuals exposed to both factors that is due to the factors’ interaction. S is the excess risk from both exposures when there is an additive interaction, relative to the risk from both exposures without an interaction. RERI = 0, AP = 0, or S = 1 means no interaction or strict additivity; RERI > 0, AP > 0, or S > 1 means positive interaction or more than additivity; RERI < 0, AP < 0, or S < 1 means negative interaction or less than additivity [32]. If any of the null values (0 in RERI and AP or 1 in S) falls outside the 95 % CI of its respective measurement, then the additive interaction is considered statistically significant. All statistical analyses were performed using STATA/SE software version 12.0 (StataCorp, College Station, TX, USA).

## Results

Compared with control subjects, cases were more likely to be older, to report never having smoked, and to report a low intake of caffeine (Table 1). There were no differences between cases and controls with regard to sex or region of residence.

**Table 1:** Characteristics of the study population

	***n*(%) or mean (SD)**		
**Variable**	**Cases (*N* = 229)**	**Controls (*N* = 357)**	***P*-value**
Sex (%)			0.90
Male	88 (38.4)	139 (38.9)	
Female	141 (61.6)	218 (61.1)	
Age, years	68.4 (8.7)	66.6 (8.5)	0.02
Onset age, years	65.7 (8.8)		
Region of residence (%)			0.28
Fukuoka	86 (37.6)	150 (42.0)	
Kinki	143 (62.5)	207 (58.0)	
Ever smoked (%)			0.001
No	167 (72.9)	212 (59.4)	
Yes	62 (27.1)	145 (40.6)	
Caffeine intake, mg/4184 kJ	149.3 (110.8)	194.0 (139.2)	< 0.0001

There was no significant deviation from the Hardy-Weinberg equilibrium in the control subjects (*P* = 0.67).

Under a recessive model, compared with subjects with the CC or CA genotype of *UCHL1 * S18Y SNP, those with the AA genotype had a significantly increased risk of sporadic PD after adjustment for sex, age, region of residence, smoking, and caffeine intake: the adjusted OR was 1.57 (95 % CI: 1.06 − 2.31) (Table 2). No such significant association was found under the co-dominant, additive, or dominant model. The frequency of the A allele was 52.0 % in cases and 47.5 % in controls; thus the allelic distribution was not significantly different between cases and controls (*P* = 0.13).

**Table 2:** **Association of*UCHL1 *S18Y polymorphism with sporadic Parkinson’s disease in a Japanese population**

		***n*(%)**			
** Model**	**Genotype**	**Cases (*N* = 229)**	**Controls (*N* = 357)**	**Crude OR (95 % CI)**	Adjusted OR (95 % CI)*
Co-dominant	CC	61 (26.6)	96 (26.9)	1.00	1.00
	CA	98 (42.8)	183 (51.3)	0.84 (0.56 − 1.26)	0.85 (0.56 − 1.30)
	AA	70 (30.6)	78 (21.9)	1.41 (0.90 − 2.23)	1.41 (0.88 − 2.27)
Additive				1.19 (0.94 − 1.50)	1.19 (0.94 − 1.51)
Dominant				1.01 (0.70 − 1.47)	1.02 (0.69 − 1.51)
Recessive				1.57 (1.08 − 2.30)	1.57 (1.06 − 2.31)

*Adjusted for sex, age, region of residence, smoking, and caffeine intake.

Based on the recessive model, compared with subjects with the CC or CT genotype of *SNCA* SNP rs356220, the TT genotype was significantly related to an increased risk of sporadic PD after adjustment for confounders: the adjusted OR was 1.47 (95 % CI: 1.03 − 2.10). Using subjects with the CC or CA genotype of *UCHL1 * S18Y SNP and the CC or CT genotype of *SNCA* SNP rs356220 as a reference group, those with the AA genotype of *UCHL1 * S18Y SNP and the TT genotype of *SNCA* SNP rs356220 had a significantly increased risk of sporadic PD; however, neither multiplicative nor additive interaction was statistically significant (Table 3).

**Table 3:** **Interaction between*UCHL1 *S18Y and*SNCA*rs356220 polymorphisms affecting sporadic Parkinson’s disease in a Japanese population**

	***SNCA *rs356220**			
	**CC + CT**		**TT**	
**Genotype**	**No. cases/controls**	Adjusted OR (95 % CI)*	**No. cases/controls**	Adjusted OR (95 % CI)*
CC + CA	92/182	1.00	67/97	1.44 (0.94 − 2.19)
AA	37/48	1.51 (0.91 − 2.53)	33/29	2.31 (1.29 − 4.14)
*P * for multiplicative interaction = 0.91				
Measures of additive interaction^†^				
Relative excess risk due to interaction (RERI) = 0.34 (95 % CI: -1.14 − 1.81)				
Attributable proportion due to interaction (AP) = 0.15 (95 % CI: -0.44 − 0.73)				
Synergy index (S) = 1.35 (95 % CI: 0.37 − 4.94)				

*Adjusted for sex, age, region of residence, smoking, and caffeine intake.

^†^Statistically significant when the 95 % CI of RERI > 0, the 95 % CI of AP > 0, or the 95 % CI of S > 1, indicating additive interaction.

Compared with ever having smoked, never having smoked was significantly related to an increased risk of sporadic PD after adjustment for sex, age, region of residence, and caffeine intake: the adjusted OR was 2.63 (95 % CI: 1.64 − 4.22). Compared with subjects with the CC or CA genotype of *UCHL1 * S18Y SNP who had ever smoked, those with the AA genotype who had never smoked had a 4.08-fold increased risk of sporadic PD; nevertheless, neither multiplicative nor additive interaction was significant (Table 4).

**Table 4:** **Interaction between*UCHL1 *S18Y polymorphism and smoking status affecting sporadic Parkinson’s disease in a Japanese population**

	**Ever smoked**			
	**Yes**		**No**	
**Genotype**	**No. cases/controls**	Adjusted OR (95 % CI)*	**No. cases/controls**	Adjusted OR (95 % CI)*
CC + CA	42/113	1.00	117/166	2.73 (1.62 − 4.61)
AA	20/32	1.72 (0.87 − 3.38)	50/46	4.08 (2.18 − 7.63)
*P * for multiplicative interaction = 0.74				
Measures of additive interaction^†^				
Relative excess risk due to interaction (RERI) = 0.63 (95 % CI: -1.48 − 2.75)				
Attributable proportion due to interaction (AP) = 0.16 (95 % CI: -0.31 − 0.62)				
Synergy index (S) = 1.26 (95 % CI: 0.59 − 2.68)				

*Adjusted for sex, age, region of residence, and caffeine intake.

^†^Statistically significant when the 95 % CI of RERI > 0, the 95 % CI of AP > 0, or the 95 % CI of S > 1, indicating additive interaction.

When caffeine intake was categorized into two groups, with the cut-off point defined as the median intake among control subjects, intake of less than 161.7 mg/4184 kJ/day was significantly associated with an increased risk of sporadic PD: the adjusted OR was 1.70 (95 % CI: 1.20 − 2.41). There was no significant multiplicative or additive interaction between *UCHL1 * S18Y SNP and caffeine intake (Table 5).

**Table 5:** **Interaction between*UCHL1 *S18Y polymorphism and caffeine intake affecting sporadic Parkinson’s disease in a Japanese population**

	**Caffeine intake**			
	**≥ 161.7 mg/4184 kJ**		**< 161.7 mg/4184 kJ**	
**Genotype**	**No. cases/controls**	Adjusted OR (95 % CI)*	**No. cases/controls**	Adjusted OR (95 % CI)*
CC + CA	58/140	1.00	101/139	1.82 (1.21 − 2.75)
AA	28/38	1.87 (1.03 − 3.36)	42/40	2.66 (1.55 − 4.59)
*P * for multiplicative interaction = 0.55				
Measures of additive interaction^†^				
Relative excess risk due to interaction (RERI) = −0.02 (95 % CI: -1.63 − 1.59)				
Attributable proportion due to interaction (AP) = −0.01 (95 % CI: -0.61 − 0.60)				
Synergy index (S) = 0.99 (95 % CI: 0.38 − 2.58)				

*Adjusted for sex, age, region of residence, and smoking.

^†^Statistically significant when the 95 % CI of RERI > 0, the 95 % CI of AP > 0, or the 95 % CI of S > 1, indicating additive interaction.

## Discussion

To our knowledge, this is the first study to find a significant positive association between the *UCHL1 * S18Y variant and the risk of sporadic PD. Our results are at variance with those from the previously cited meta-analysis, which demonstrated a significant inverse association between the Y allele and PD [5]. The PDGene meta-analysis in the website (http://www.pdgene.org) demonstrated that the Y allele of *UCHL1 * S18Y SNP was significantly inversely related to PD using data from a total of 26 case–control samples; however, such a significant association was not observed using data from 12 case–control Asian samples [33]. Zhang et al. [6] showed that the frequency of the Y allele of *UCHL1 * S18Y SNP was significantly higher among control subjects than among PD patients in a Japanese population (crude OR = 0.73 [95 % CI: 0.53 − 0.99]), whereas, under a recessive model, the AA genotype was non-significantly positively associated with the risk of sporadic PD in a Caucasian population (crude OR = 4.76 [95 % CI: 0.52 − 226.8]); furthermore, the frequency of the AA genotype was much lower among Caucasians than among Japanese. A case–control study of Australians found a non-significant positive association between the AA genotype of *UCHL1 * S18Y SNP and PD under a recessive model (crude OR = 3.14 [95 % CI: 0.76 − 18.32]) [13]. The current results are in partial agreement with these results from Caucasian populations. With regard to the other case–control studies of Japanese, one study showed a significant inverse association between the AA genotype of *UCHL1 * S18Y SNP and PD [7], while such a significant association was not found in two other studies [9,25] (Table 6). The frequency of the AA genotype of *UCHL1 * S18Y SNP was lower among our control subjects than among control subjects in all of the previous studies of Japanese [6,7,9,25]. The inconsistency of our findings with those of other studies may be at least partly explained by differences in the genetic backgrounds of the populations examined, definitions of PD, and statistical power. In a genome-wide association study of Japanese, there was no relationship between *UCHL1 * S18Y SNP and PD at a genome-wide significance level; however, about 64 % of control subjects were those registered in BioBank Japan as subjects with diseases other than a neurological disease, and the mean age of the control subjects was 49.9 [34].

**Table 6:** Genotype and allele frequencies from previous case–control studies of Japanese populations investigating the association between*UCHL1 *S18Y polymorphism and Parkinson’s disease*

	**Zhang et al. [**[6]**]**		**Momose et al. [**[7]**]**		**Mizuta et al. [**[9]**]**^**†**^		**Snapinn et al. [**[25]**]**	
	**Cases**	**Controls**	**Cases**	**Controls**	**Cases**	**Controls**	**Cases**	**Controls**
**Genotype**	***N* = 160**	***N* = 160**	***N* = 230**	***N* = 248**	***N* = 613**	***N* = 736**	***N* = 605**	***N* = 1620**
CC	52 (32.5)	35 (21.9)	71 (30.9)	61 (24.6)	149 (24.3)	199 (27.0)	150 (24.8)	412 (25.4)
CA	77 (48.1)	86 (53.8)	119 (51.7)	122 (49.2)	340 (55.5)	366 (49.7)	313 (51.7)	805 (49.7)
AA	31 (19.4)	39 (24.4)	40 (17.4)	65 (26.2)	124 (20.2)	171 (23.2)	142 (23.5)	403 (24.9)
**Allele**	***N* = 320**	***N* = 320**	***N* = 460**	***N* = 496**	***N* = 1226**	***N* = 1472**	***N* = 1210**	***N* = 3240**
C	181 (56.6)	156 (48.8)	261 (56.7)	244 (49.2)	638 (52.0)	764 (51.9)	613 (50.7)	1629 (50.3)
A	139 (43.4)	164 (51.3)	199 (43.3)	252 (50.8)	588 (48.0)	708 (48.1)	597 (49.3)	1611 (49.7)

* *n* (%).

^†^Data were based on a meta-analysis by Ragland et al. [5]; excluded cases included in genotype counts in the Momose et al. study [7].

Andersson et al. [35] have shown that the S18Y form of UCHL1 is minimally perturbed compared to the wild type. Although the primary effect of the mutation is to slightly reduce stability, the structure is barely perturbed, as shown by fluorescence, far-UV CD, and NMR measurements: they have found no evidence that would suggest that S18Y may behave in such a way as to contribute to a decreased risk of PD [35]. We are unable to explain why we found a significant positive association between the *UCHL1 * S18Y variant and the risk of sporadic PD.

Co-localization of UCHL1 and α-synuclein proteins in nigral Lewy bodies has been shown in human sporadic PD brain, suggesting a possible physical and/or functional interaction between the two proteins [26]. In the current study, however, no significant interaction was found between *UCHL1 * S18Y and *SNCA* SNP rs356220 with respect to the risk of sporadic PD. With regard to gene-environment interactions, there were no significant interactions between *UCHL1 * S18Y and smoking or caffeine intake affecting the risk of sporadic PD; however, subjects with the AA genotype who had never smoked had a 4.08-fold increased risk of sporadic PD. A study of US individuals observed that *UCHL1 * and UCHL1 at the protein level were up-regulated in smokers compared with nonsmokers [36]. A case–control study using data from the NeuroGenetics Research Consortium found no significant interactions between *UCHL1 * S18Y and smoking or coffee intake with respect to the risk of PD [37].

Several limitations of our study warrant mention. First, data on family history of PD were self-reported; accordingly, it is assumed that some proportions of sporadic PD may be misclassified. Second, data on smoking and caffeine intake were also self-reported. Third, the current study size was rather small for a valid genetic association study. Although a significant positive relationship between *UCHL1 * S18Y SNP and sporadic PD was observed, the insignificant additive interactions under study might be attributable to insufficient statistical power. Fourth, correction for multiple testing was not performed in the present study because this is a hypothesis testing study. Fifth, although adjustment was made for some confounders, residual confounding effects could not be ruled out.

## Conclusions

The results of the current Japanese case–control study reveal that the AA genotype of *UCHL1 * S18Y is significantly associated with an increased risk of sporadic PD under a recessive model. Nevertheless, we could not find evidence for interactions between *UCHL1 * S18Y and *SNCA* SNP rs356220, smoking, or caffeine intake with regard to the risk of sporadic PD. We acknowledge that our findings must be confirmed by further case–control studies with a larger number of subjects and also by functional studies.

## Appendix

Other members of the Fukuoka Kinki Parkinson’s Disease Study Group are as follows: Yasuhiko Baba and Tomonori Kobayashi (Department of Neurology, Faculty of Medicine, Fukuoka University); Hideyuki Sawada, Eiji Mizuta, and Nagako Murase (Clinical Research Institute and Department of Neurology, Utano National Hospital); Tsuyoshi Tsutada and Takami Miki (Department of Geriatrics and Neurology, Osaka City University Graduate School of Medicine); Jun-ichi Kira (Department of Neurology, Neurological Institute, Graduate School of Medical Sciences, Kyushu University); Tameko Kihira and Tomoyoshi Kondo (Department of Neurology, Wakayama Medical University); Hidekazu Tomimoto (Department of Neurology, Kyoto University Graduate School of Medicine); Takayuki Taniwaki (Division of Respirology, Neurology, and Rheumatology, Department of Medicine, Kurume University School of Medicine); Hiroshi Sugiyama and Sonoyo Yoshida (Department of Neurology, Minami-Kyoto National Hospital); Harutoshi Fujimura and Tomoko Saito (Department of Neurology, Toneyama National Hospital); Kyoko Saida and Junko Fujitake (Department of Neurology, Kyoto City Hospital); Naoki Fujii (Department of Neurology, Neuro-Muscular Center, National Omuta Hospital); Masatoshi Naito and Jun Arimizu (Department of Orthopaedic Surgery, Faculty of Medicine, Fukuoka University); Takashi Nakagawa, Hirofumi Harada, and Takayuki Sueta (Department of Otorhinolaryngology, Faculty of Medicine, Fukuoka University); Toshihiro Kikuta and George Umemoto (Department of Oral and Maxillofacial Surgery, Faculty of Medicine, Fukuoka University); Eiichi Uchio and Hironori Migita (Department of Ophthalmology, Faculty of Medicine, Fukuoka University); Kenichi Kazuki, Yoichi Ito, and Hiroyoshi Iwaki (Department of Orthopaedic Surgery, Osaka City University Graduate School of Medicine); Kunihiko Siraki and Shinsuke Ataka (Department of Ophthalmology and Visual Sciences, Osaka City University Graduate School of Medicine); Hideo Yamane and Rie Tochino (Department of Otolaryngology and Head and Neck Surgery, Osaka City University Graduate School of Medicine); Teruichi Harada (Department of Plastic and Reconstructive Surgery, Osaka City University Graduate School of Medicine); Yasushi Iwashita, Motoyuki Shimizu, Kenji Seki, and Keiji Ando (Department of Orthopedic Surgery, Utano National Hospital).

## Abbreviations

AP, Attributable proportion due to interaction; CI, Confidence interval; OR, Odds ratio; PD, Parkinson’s disease; RERI, Relative excess risk due to interaction; S, Synergy index; UCHL1, Ubiquitin carboxy-terminal hydrolase L1.

## Competing interests

All authors declare that they have no competing interests.

## Authors’ contributions

YM contributed to the study design, data collection, overall management, statistical analysis, data interpretation, and writing of the manuscript. KT and WF contributed to the study design, data collection, and data management. SS and CK contributed to the study design. YT, TY, TO, TM, NK, NS and HF contributed to the outcome definition and case recruitment. YH and MN contributed to the supervision of the design and execution of the study. Authors listed in the Appendix contributed to case or control subject recruitment. All authors contribute to the preparation of the manuscript and approved the final version submitted for publication.

## Pre-publication history

The pre-publication history for this paper can be accessed here:

http://www.biomedcentral.com/1471-2377/12/62/prepub
